# The Bioinformatic and In Vitro Studies of Clostridioides Difficile Aminopeptidase M24 Revealed the Immunoreactive KKGIK Peptide

**DOI:** 10.3390/cells9051146

**Published:** 2020-05-07

**Authors:** Katarzyna Pacyga, Agnieszka Razim, Gayane Martirosian, Małgorzata Aptekorz, Andrzej Szuba, Andrzej Gamian, Andrzej Myc, Sabina Górska

**Affiliations:** 1 Department of Microbiology, Hirszfeld Institute of Immunology and Experimental Therapy, Polish Academy of Sciences, 53-114 Wroclaw, Poland; katarzyna.pacyga@hirszfeld.pl; 2 Department of Immunology of Infectious Diseases, Hirszfeld Institute of Immunology and Experimental Therapy, Polish Academy of Sciences, 53-114 Wroclaw, Poland; andrzej.gamian@hirszfeld.pl (A.G.); myca@umich.edu (A.M.); 3 Department of Medical Microbiology, School of Medical Science in Katowice, Medical University of Silesia, 40-752 Katowice, Poland; gmartir@sum.edu.pl (G.M.); maptekorz@sum.edu.pl (M.A.); 4 Division of Angiology, Wroclaw Medical University, 51-618 Wroclaw, Poland; andrzej.szuba@umed.wroc.pl; 5 Department of Internal Medicine, 4th Military Hospital in Wroclaw, 50-981 Wroclaw, Poland; 6 MNIMBS, Department of Internal Medicine, University of Michigan, Ann Arbor, MI 48109-5648, USA

**Keywords:** *Clostridioides difficile*, infection, vaccine, conjugate

## Abstract

*Clostridioides difficile* (CD) is a Gram-positive pathogen responsible for CD-associated disease (CDAD), which is characterized by symptoms ranging from mild diarrhea to pseudomembranous colitis. This work is an attempt to respond to the need of novel methods for CD infection (CDI) prevention, since the number of CDI cases is still rising. A bioinformatics approach was applied to design twenty-one peptides consisting of in silico predicted linear B-cell and T-cell epitopes of aminopeptidase M24 from CD. These peptides were mapped for epitopes exploiting PEPSCAN procedure and using sera obtained from CD infected patients, umbilical cord blood, and healthy volunteers. Two new CD epitopes, ^131^KKGIK^135^ and ^184^KGTSTHVIT^192^, were identified and characterized. Immunoreactivity of the synthetic biotinylated ^131^KKGIK^135^ epitope was significantly higher compared to ^184^KGTSTHVIT^192^ epitope in Enzyme-Linked Immunosorbent Assay (ELISA) with umbilical cord blood and CDI patients’ sera. Hereafter, the conjugate of bovine serum albumin and epitope ^131^KKGIK^135^ was evaluated in vitro on lung epithelial cell line. In vitro, a significant induction of IL-6 by conjugate was observed, thereby we postulate that this new ^131^KKGIK^135^ epitope possesses immunostimulating properties suggesting possibility of its use in a vaccine against *Clostridioides difficile*.

## 1. Introduction

CD is a Gram-positive, spore-forming bacterium that grows under anaerobic conditions. It is commonly associated with CDAD occurring in hospitals and nursing homes [1,2,3,4]. CDI spreads by the fecal-oral route, mainly through spores resistant to high temperature, disinfectants, and antibiotics [5]. These spores can be found in small amounts in the soil, food, and water. Mostly, they occur in the hospital environment, covering floors and hospital equipment, like beds and cabinets [5,6]. CD may be a constituent of a healthy human gut flora [7], but it starts to overgrow when an imbalance in the intestinal microflora occurs, especially during antibiotic therapy [3,8,9,10]. CDAD varies from mild diarrhea to pseudomembranous colitis, which may lead to the death of the patient [2,6,8,11,12,13,14]. An increasing number of patients suffering from CDI, as well as a progressive mortality, have been recently noticed. This rapid increase in CDAD severity was associated with the activity of hypervirulent CD NAP1/027 strain [2,15,16], which produces greater amounts of toxins A and B (responsible for the development of the disease) than other CD strains and shows higher antibiotic resistance [1,5,17,18].

The diversity of symptoms caused by CD is a result of the individuals’ immune response to CD toxins, as well as its other proteins, such as adhesins [8]. There are several categories of the infection status that can be distinguished: healthy stage, asymptomatic carriage, acute course, convalescent stage, and relapse of the infection [11]. Healthy stage applies to people who have never had contact with CD antigens, yet they often exhibit natural immunity to its toxins [4]. This phenomenon could be caused by the preceding contact with other *Clostridium* species, for example, *C. sordelli* and its cross-linking antigens [3,4,9,11,19,20]. In addition, immunoglobulin G (IgG) antibodies against CD can be found in the umbilical cord blood [21]. Newborns have natural passive immunity, which is a result of an active transfer of these immunoglobulins through the placenta from the mother to the fetus. Asymptomatic carriage means CD colonization with no signs of the disease. These patients have higher level of specific anti-CD IgG and IgA antibodies [2,9,22]. The group of asymptomatic carriers includes 5–15% of healthy adults, of which 57% are people living in long-term care facilities [15]. Patients infected with CD who did not develop a sufficiently strong humoral anti-toxin pre-infection response show symptoms of the disease. This group can be divided into two subgroups: those who experienced only one, acute episode of CDI and recovered (convalescents), and those who suffer from relapses [3]. Compared with asymptomatic carriage, both groups, during acute CDI and relapse, demonstrate weaker immune response that manifests itself mainly by lower levels of anti-CD toxin IgG antibodies. However, in the group of first acute CDI episode, the immune response against CD toxins is still stronger than during the relapses [3,9,23,24]. To sum up, it appears that high levels of serum IgA and IgG antibodies against CD toxins, in particular IgG1 and IgG2 type, protect against CDI [3,11].

The recognition of CD toxins and non-toxic CD antigens by human immune system have an impact on the course of the infection and bacteria survival [25]. Non-toxic antigens include cell wall proteins (CWPs), like S-layer proteins (SLPs), that are responsible for adhesion to the host intestine epithelial cells, as well as for other functions crucial for bacterial virulence [7,26]. CWPs are necessary for effective colonization, leading to development of the disease [7,25,27,28,29]. Drudy et al. noticed that anti-SLP IgM level of antibodies in the sera of relapsing patients was significantly lower on the third day of infection as compared to patients having the first episode of CDI. Based on these results, the authors concluded that the presence of specific anti-SLP IgM antibodies in patient serum is associated with a decreased risk of CDI-associated diarrhea [28]. Mulligan et al. emphasized the importance of IgA antibodies detected in patients’ sera, both against toxins and non-toxic antigens [30]. Surface proteins can activate and modulate the immune response. For example, CD SLPs induce the production of proinflammatory cytokines (IL-1β, IL-6), as well as anti-inflammatory and regulatory IL-10 by monocytes [31].

The use of CD non-toxin antigens gives an advantage in fighting the infection since they are often implicated in the colonization step which is the first stage of infection. Taking all of the above into consideration, the present study was intended to indicate new immunoreactive proteins (protein M24) which can constitute the potential components of peptide vaccine. Another goal was to map the epitopes of peptidase M24 using bioinformatics approaches and identify a candidate for an epitope-based peptide vaccine. Twenty-one peptides were synthesized using PEPSCAN method followed by the evaluation of their immunoreactivity using Enzyme-Linked Immunosorbent Assay (ELISA). Three groups of sera: sera from patients during first CDI episode, healthy individuals, and umbilical cord blood sera were employed in the test. The ^131^KKGIK^135^ peptide conjugated with a bovine serum albumin carrier protein (BSA) was examined for its immunostimulatory properties in vitro. The aim of the study was to assist future efforts to develop an effective vaccination to prevent CDI.

## 2. Materials and Methods

### 2.1. Human Sera

CDI patients involved in the study (*n* = 15) were diagnosed based on the following symptoms: three or more loose stools within 24 h, fever, abdominal pain, and positive test results for the presence of glutamate dehydrogenase, as well as CD toxins (C. Diff Quik Chek Complete; TECHLAB, Inc., Blacksburg, VA, USA) [6]. Healthy volunteers’ sera (*n* = 10) were obtained from the Military Blood Donors Center in Wroclaw. Human umbilical cord blood sera (*n* = 10) were collected from healthy female patients of the Obstetric Clinic of the Medical University of Wroclaw. Within groups, the sera were pooled, aliquoted, and stored at −20 °C until further used.

A written informed consent was obtained from each patient. Written approval for the use of all above described sera in this research was delivered by the Bioethics Committee of the Medical University of Wroclaw (no. KB-631/2015). Experiments were conducted in accordance with the Helsinki Declaration, 1975.

### 2.2. Identification of Immunoreactive Peptidase M24

#### 2.2.1. Bacterial Strain

A Cd27 strain isolated from a patient with CDI was used in this research (Polish Collection of Microorganisms as PCM2827). The strain was cultured in BHI (Brain Heart Infusion) + 0.05% L-cysteine in anaerobic chamber (A35 Whitley anaerobic Workstation, Bingley, UK) at 37 °C, for 48 h. Bacterium identity was confirmed using ANC cards in an automatic VITEK 2 Compact system (bioMérieux, Marcy L’Etoile, France). Toxin production (TcdA, TcdB, and binary toxin) was confirmed using the previously described methods [32]. 

#### 2.2.2. Surface Proteins Isolation

Surface proteins isolation was performed as previously published [21,33,34]. Shortly, bacterial mass was incubated with 1 M LiCl at room temperature (RT) for 30 min with shaking. Then, remaining bacteria were centrifuged at 6000× g for 5 min (Heraeus Centrifuge Stratus, Thermo Fisher Scientific, Waltham, MA, USA). The supernatant was collected and dialyzed to miliQ water for 48 h and concentrated using Amicon Ultra MWCO = 10,000 Da (Thermo Fisher Scientific). The protein content was measured using Pierce BCA Protein Assay Kit (Thermo Fisher Scientific) as in the manufacturer’s protocol. Protein samples were aliquoted and stored until further used at −20 °C.

#### 2.2.3. SDS-PAGE Electrophoresis

Thirty micrograms of CD surface proteins were loaded on two 12.5% polyacrylamide gels (dimensions of 18 × 16 cm, 1.5 mm thickness). Gels were resolved in SE600 Standard Dual Cooled Vertical Unit (Hoefer, Holliston, MA, USA) in electrophoresis buffer (0.025 M Tris, 0.192 M glycine and 0.1% SDS). Prior to the electrophoresis, protein samples were suspended in Laemmli buffer [35] and denatured at 95 °C for 5 min. A protein mass marker (Precision Plus Dual Color, Bio-Rad, Hercules, CA, USA) was used. Electrophoresis was run for 5 h in 150 V with cooling. One of the duplicated gels was used for Western blotting, and the second was silver-stained and used for protein identification.

#### 2.2.4. Western Blotting

Western blotting was used to test the immunoreactivity of isolated CD surface proteins [36] and was performed as previously described by us [34]. Briefly, proteins were transferred on the Immobilon P membrane (Merck Burlington, MA, USA), which was then blocked with BSA and incubated with umbilical cord blood serum (in the dilution of 1:100 in 0.1% BSA in tris-buffered saline with the addition of 0.05% Tween 20 (TBS-T buffer). After overnight incubation, the membrane was washed and immersed in anti-human IgG antibodies conjugated with alkaline phosphatase (cat. A1543-1ml, Sigma-Aldrich, Saint Louis, MO, USA), in the dilution of 1:10 000 in TBS-T buffer for 1 h at RT with shaking. The membrane was washed and the color reaction was developed by adding nitro blue tetrazolium/5-bromo-4-chloro-3-indolyl phosphate (NBT/BCIP) solution (Sigma-Aldrich). The reaction was stopped by multiple washing in water. The membrane was documented with Gel Doc™ (Bio-Rad).

#### 2.2.5. Silver Staining

The duplicated SDS-PAGE electrophoresis gel was stained by silver method as described previously [37]. Protein bands were cut out directly after gel staining to avoid any contamination. After that, the gel was documented with Gel Doc™.

#### 2.2.6. Protein Identification

Immunoreactive bands identified by antibodies from umbilical cord blood sera in Western blotting were compared to silver-stained bands. An immunoreactive band of a molecular mass ca. 68 kDa was cut out from the gel. Then, the proteins were digested with trypsin, and subsequent peptides were separated by liquid chromatography and identified by LC-MS-MS/MS Orbitrap. The obtained peptides of different masses were compared with those collected in NCBI and UniProt databases by using MASCOT program (http://www.matrixscience.com/). All searches were done looking for proteins from the *Peptoclostridium difficile*.

### 2.3. Bioinformatic Analysis of Peptidase M24

#### 2.3.1. Analysis of M24 Peptidase Amino Acid Sequence

The National Center for Biotechnology Information (NCBI, https://www.ncbi.nlm.nih.gov/) [38] and UniProt (https://www.uniprot.org/) [39] databases were used to gather 49 amino acid sequences of CD peptidase M24 homologues. These were divided into four groups: proteins from species belonging to genus *Clostridium* (16; Table S1); human proteins (2; Table S2); proteins from human commensal flora (18; Table S3); and proteins from CD strains (13; Table S4). Afterwards, they were aligned with the reference protein sequence of CD R20291 strain (the input sequence was CBE05263.1) using online aligning tools Clustal Omega (https://www.ebi.ac.uk/Tools/msa/clustalo/) [40] and Basic Local Alignment Search Tool (BLAST, https://blast.ncbi.nlm.nih.gov/Blast.cgi).

#### 2.3.2. Modeling of M24 Peptidase Protein Structure and Predicting Its Subcellular Localization

The structural model of peptidase M24 from CD was created by SWISS-MODEL Workspace (https://swissmodel.expasy.org/) [41]. It was based on the protein sequence with the highest homology to peptidase M24 and with known crystal structure. It was human cytosolic X-prolyl Xaa-Pro aminopeptidase 1 (template 3ctz.1.A, obtained by X-ray diffraction at 1.60Å resolution). The homology was 39.73%. Obtained structural model of peptidase M24 was used later in search for potential epitopes and localizing the final epitopes.

Additionally, Bologna Unified Subcellular Component Annotator (BUSCA; http://busca.biocomp.unibo.it/) [42], PSORTb (https://www.psort.org/psortb/) [43], and Gpos-mPLoc (http://www.csbio.sjtu.edu.cn/bioinf/Gpos-multi/) [44] online tools were used to predict subcellular localization of peptidase M24.

#### 2.3.3. In Silico Prediction of M24 Peptidase Epitopes

First, referring to the created peptidase M24 structural model, free loops were identified. Subsequently, based on the CD R20291 peptidase M24 sequence, potential epitopes for B and T-cells were predicted in silico. The prediction was performed only for the most frequently occurring alleles recommended by online tools that were used in the study: Immune Epitope Database and Analysis Resource (IEDB; https://www.iedb.org) with the threshold set to 0.5 for B-cell epitopes [45], Support Vector Machine to Integrate Tri-Peptide Similarity and Propensity (SVMTriP; http://sysbio.unl.edu/SVMTriP/) that indicated recommended results [46], and TepiTool (a part of IEDB database; http://tools.iedb.org/tepitool/) with cutoff IC50 results value above 500 nm [47]. Only epitopes with minimum length of 3 amino acids were taken into further consideration.

#### 2.3.4. The Analysis of Structure and Solvent Accessibility of Epitopes

The structural analysis of epitopes was performed using PEP-FOLD3 (http://bioserv.rpbs.univ-paris-diderot.fr/services/PEP-FOLD3/) [48]. The program allows spatial visualization of sequences with length of 5 to 50 amino acids, which is especially important in studies focused on short peptide sequences. Swiss-PDBb-Viewer [49] was used for the visualization of selected peptides.

Additionally, solvent accessibility of studied sequences was checked in sequence-based NetSurfP-2.0 software (http://www.cbs.dtu.dk/services/NetSurfP/) and PDB model based CUPSAT program (http://cupsat.tu-bs.de/).

#### 2.3.5. Searching for Autoimmunoreactivity

Reactive peptides were checked for autoimmune attributes by IEDB and HMMER (https://www.ebi.ac.uk/Tools/hmmer/search/phmmer) [50].

### 2.4. Epitope Mapping

#### 2.4.1. Epitope Synthesis

The synthesis of selected epitopes was carried out on the hydroxypropyl methacrylic pins (noncleavable peptide type, MIMOTOPES, Melbourne, Australia) under RT conditions based on the Geysen et al. [51] procedure modified by Jarząb et al. [52]. Coupling was performed by subsequent addition of F-moc amino acids derivatives with blocked side groups (MIMOTOPES). Synthesis consisted of two major steps: pins deprotection in 20% piperidine in dimethylformamide (DMF, SupraSolv, Burlington, MA, USA) and the acylation reaction performed on polyethylene 96-well plates. The coupling reaction was performed in an acylation solution (60 mM Fmoc amino acids derivatives (MIMOTOPES), 65 mM 1-hydroxy-7-azabenzotriazole (Sigma-Aldrich), 60 mM diisopropylcarbodiimide (Merck,), bromophenol blue in DMF) for at least 6 h in a sealed vessel. This procedure was repeated until the last amino acid was added to the pin. After obtaining a full-length peptide, the pins were deprotected in an unblocking cocktail (2.5% anisole (Sigma-Aldrich), 2.5% ethanedithiol (Fluka Analytical, Charlotte, NC, USA) in trifluoracetic acid (Merck). Finally, the pins were washed successively by methanol, followed by 0.5% solution of acetic acid in methanol and miliQ (1:1) and again in methanol. Then, the pins were left to dry and stored at −20 °C until used. 

#### 2.4.2. ELISA with Pin-Bound Peptides

Immunoreactivity of pin-bound synthesized epitopes was tested at RT by ELISA [51,53] against three groups of pooled sera: initial CDI episode, umbilical cord blood, or healthy volunteers. The assay consisted of four main steps: pins incubation with: (1) blocking solution containing 1% BSA (SeraCare, Milford, MA, USA) in TBS-T; (2) primary antibodies in a 1:1000 dilution in TBS-T with 0.1% BSA in TBS-T; (3) secondary antibodies – anti-human IgG conjugated with AP (cat. A1543-1ml, Sigma-Aldrich) in a 1:10000 dilution in TBS-T; and (4) Alkaline Phosphatase Yellow Liquid Substrate System for ELISA (AP Yellow, Sigma-Aldrich). Plates were read at 405 nm (PowerWave HT, BioTek Instruments, Winooski, VT, USA). After assay, bound antibodies were stripped off by sonication in disruption buffer (1% sodium dodecyl sulfate, 0.1% 2-mercaptoethanol and 0.1 M Na_3_PO_4_) preheated to 60 °C, washed in water and methanol, and left to dry. 

Based on the results obtained in ELISA assays, the baseline was calculated, and peptides were selected for further analysis. In his work, Carter suggested that the background should be calculated as the mean of the 10–25% of the lowest results [51]. We, however, considered the baseline as a mean of the results obtained for all peptides, which is a more restrictive approach.

#### 2.4.3. Finding the Functional Epitope

To determine the shortest immunoreactive sequences, the Window net (or “Wnet”) strategy was used [54]. The procedure is based on gradual shortening of selected peptides and analysis of their changing immunoreactivity. The most reactive 16-amino acid peptides were synthesized again on the hydroxypropyl methacrylic pins in truncated variants (up to 4 amino acids left), in which every amino acid was successively subtracted from the C- or the N-terminus. Then, obtained variants were tested by ELISA to define the shortest immunoreactive peptides. Furthermore, to identify which amino acids in the epitope are responsible for interaction with the antibody (functional epitope), tested peptides were re-synthesized using the alanine-walk method [55]. In a tested peptide each amino acid was substituted by alanine. The level of immunoreactivity of substituted peptides was assessed by ELISA. 

#### 2.4.4. ELISA with Synthetic Biotinylated Peptides

Biotinylated peptides (biotin-Ttds-NH2 peptides) were obtained from JPT Peptide Technologies (JPT, Berlin, Germany). ELISA assay with biotinylated peptides was performed on Pierce^®^ Streptavidin Coated 96-Well Plates (Thermo Fisher Scientific) according to the manufacturer’s procedure with slight modifications. Biotinylated peptides were added to the plate in a concentration of 10 µg/mL in 0.1 M carbonate buffer (pH = 9.5). All the reaction components were used as in 2.4.2. method apart from secondary antibodies (S3821, Promega, AP-conjugated, dilution 1:10000). The colorimetric reaction was stopped with 3 M NaOH solution. Results were read at 405 nm using the plate reader. 

### 2.5. In Vitro Cell Stimulation Experiment

#### 2.5.1. Cell Line and Culture

The Tissue Culture number one (TC-1) cell line (ATCC^®^ CRL-2785™, Manassas, VA, USA), which is an immortalized epithelial cell line from a mouse model of lung cancer, was obtained from the cell line collection of the Hirszfeld Institute of Immunology and Experimental Therapy of the Polish Academy of Sciences. TC-1 cells were cultured on Dulbecco’s Modified Eagle Medium (DMEM, Gibco, Thermo Fisher Scientific) with fetal bovine serum (FBS; 10% final concentration, Gibco), L-Glutamine-Penicillin-Streptomycin solution (1% final concentration, Sigma-Aldrich, St. Louis, MO, USA). This cell line is adherent, the passage was carried out at the time when cells covered 80 to 90% of the culture bottles’ surface (every second day). Cells were cultured in an incubator (Binder, Tuttlingen, Germany) at 37 °C with 5% CO_2_ and appropriate humidity.

#### 2.5.2. TC-1 Cell Line Stimulation with KKGIKC-BSA

The conjugate KKGIKC-BSA was obtained by the use of the thiol maleimide chemistry (JPT). It involves adding an extra cysteine residue containing thiol group that allows coupling with the maleimide modified carrier protein at the end of the peptide [56,57]. The delivered conjugate was analyzed by HPLC and MS techniques, which showed over 85% purity and about 8–9 peptide units per BSA molecule. The purified conjugate was subjected to the Limulus Amoebocyte Lysate (LAL) assay (Pierce^®^ LAL Chromogenic Endotoxin Quantitation Kit; Thermo Fisher Scientific). LAL assay was performed according to the manufacturer’s procedure.

In addition, a conjugate of BSA and irrelevant peptide (VKEFRVATGKK, epitope of immunoreactive CD protein Cwp22 [34]) prepared identically as conjugate KKGIKC-BSA was used as a control.

Stimulation of TC-1 cell line was carried out in order to test the cellular response to the KKGIKC-BSA conjugate. Cells were seeded on a 24-well TC-treated plate (0.2 × 10^6^ cells/well). Next day, they were stimulated with KKGIKC-BSA, VKEFRVATGKKC-BSA, KKGIK peptide and BSA alone at the concentration of 10 µg/mL. Culture medium was used as a negative control and lipopolysaccharide (LPS; *Escherichia coli* 055:B5 cat. L6529 Sigma-Aldrich) as a positive control. After another 24 h, cell culture supernatants were collected from each well and kept at −20 °C until used. Collected supernatants were analyzed using Mouse IL-6 DuoSet ELISA (R&D Systems, Minneapolis, MN, USA) according to manufacturer’s instructions.

### 2.6. Statistical Analysis

All experiments were repeated at least three times, SD was calculated and data were analyzed with 1- or 2-way ANOVA with Bonferroni posttest or Dunnett’s multiple comparisons. Cell line stimulation results were tested with unpaired t-test. All statistical analysis and visualizations of obtained data were prepared with the use of Graph Pad Prism version 8.

## 3. Results

### 3.1. The M24 Peptidase Identified as One of the Immunoreactive Proteins of CD

**Figure 1:**
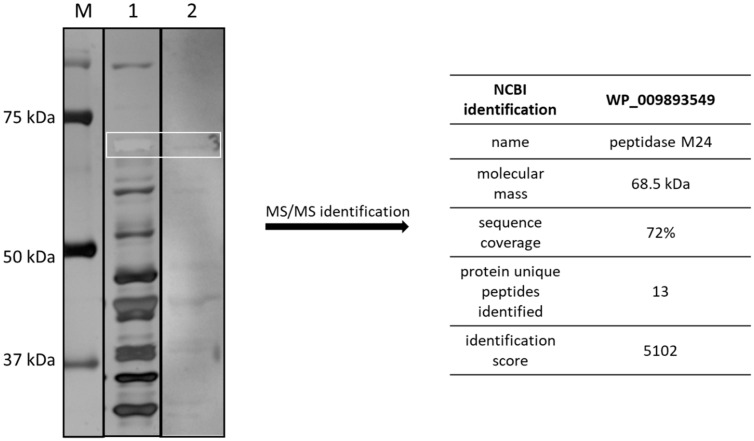
Identification of M24 as a one of the immunoreactive proteins of CD. Surface protein isolate was separated using SDS-PAGE electrophoresis (12.5% gel). One gel was stained with silver method (lane 1). The duplicate gel was subjected to Western blotting using umbilical cord blood serum as primary antibodies and anti-human IgG as secondary antibodies (lane 2). Immunoreactive band (white box) was excised from the gel and identified by MS/MS analysis. M-marker lane. NCBI = National Center for Biotechnology Information.

CD proteins which were isolated using gentle washing-off method designed for the isolation of surface proteins were separated using 1D SDS-PAGE electrophoresis and subjected to Western blotting analysis with umbilical cord blood serum as primary antibody (Figure 1). Immunoreactive protein band of molecular mass of c.a. 69 kDa was cut out of the gel and identified by mass spectrometry as peptidase M24. There were 13 unique peptides identified and the sequence coverage was 72% which confirms reliable identification. Some of the other immunoreactive proteins shown on Figure 1 were also identified and characterized by us in previous articles [21,34].

### 3.2. M24 Peptidase Remains Conservative Among Clostridioides Difficile Strains

In order to determine the prevalence of the M24 amino acid sequence in the environment, M24 amino acid sequence of CD R20291 was aligned with its homologues (49 sequences) from other organisms using Clustal Omega and BLAST. The peptidase M24 sequence shows low similarity to its homologues in humans (35–40%) (Table S1), while in human flora it varies from 23% in *Bifidobacterium animalis* subsp. *lactis* to 86% in *Enterococcus faecalis* (Table S2). Similarity increases within other *Clostridium* species (up to 73% for *Clostridium dakarense*) (Table S3) and is the highest within *Clostridioides difficile* strains (up to 100%) (Table S4).

### 3.3. Peptidase M24 Structural Model

**Figure 2:**
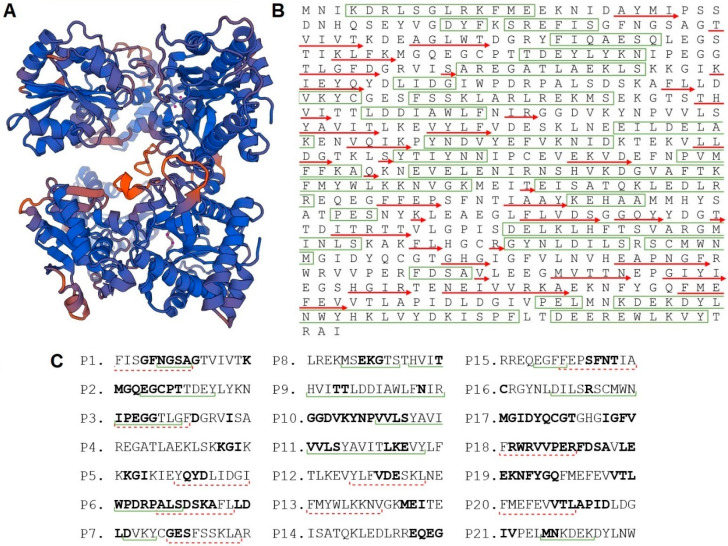
Results of the bioinformatics analysis of peptidase M24. (**A**) Structural model of peptidase M24 created by SWISS-MODEL. (**B**) Localization of α-helices (green rectangles), β-structures (red arrows) in the protein sequence. (**C**) Peptides selected for PEPSCAN synthesis with underlined B-cell (green, continuous line) and T-cell (red, dotted line) in silico predicted epitopes. Bold amino acids correspond with unstructured protein sequences.

The crystallographic structure of peptidase M24 is unknown, so a structural model of CD M24 was created by us using SWISS-MODEL Workspace on the basis of the template proposed by HHHBlits (template 3ctz.1.A). The used template is human cytosolic X-Prolyl Xaa-Pro aminopeptidase (39.73% homology to M24). According to our model, peptidase M24 is a homodimer in which ligands are four Mn^2+^ ions. It consists of 38.86% α-helix, 20.77% β-strands, and 40.37% loops (Figure 2A,B). The QMEAN value (the Qualitative Model Energy Analysis), which consists of several separate factors, was estimated, and it reached −2.49 for obtained structural model. Results between −4 and 0 show that the model created by the program is of good quality; for that reason, it was used later in epitope mapping. The prediction of the protein localization showed that peptidase M24 is predominantly localized in the cytosol.

### 3.4. Peptidase M24 Contains Immunoreactive Peptides

**Figure 3:**
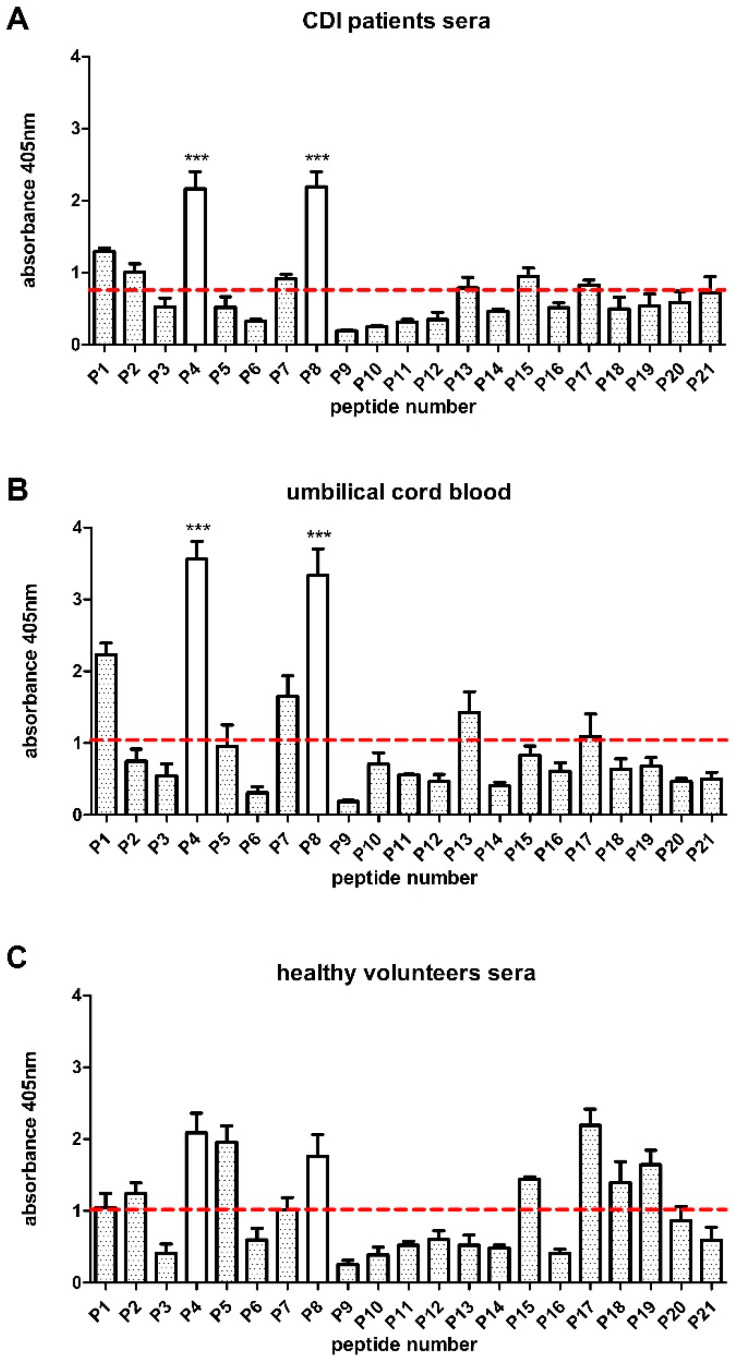
Immunoreactivity of pin-bound peptides with tested groups of sera: (**A**) CDI patients’ sera; (**B**) umbilical cord blood sera; (**C**) healthy volunteers’ sera. Sera antibodies (used in dilution of 1:1000) bound to epitopes were recognized by anti-human IgG secondary antibodies conjugated with AP. Colorimetric reaction was developed with AP ellow Substrate and the result was measured spectrophotometrically at λ = 405 nm. ELISA was repeated at least three times. The dotted line is a counted baseline. Data are shown with mean and ±SD. 2-way ANOVA and Bonferroni posttest were performed. Significant differences were counted for each peptide comparing to the baseline (*** *p* ≤ 0.001).

Analysis of the obtained structural model together with bioinformatics prediction of B-cell and T-cell epitopes enabled the selection of 21 peptides, all of them are 16 amino acids long (Figure 2C). These sequences were synthesized using PEPSCAN procedure and, subsequently, their immunoreactivity was analyzed by pin-bound peptide ELISA using pooled CDI patient sera, healthy volunteers’ sera, or umbilical cord blood sera (Figure 3). In order to select peptides for further research, a baseline (mean absorbance of all peptides) was calculated, and statistically significant differences between the peptides and the baseline were determined. Results indicated that P4 (^120^REGATLAEKLSKKGIK^135^) and P8 (^177^LREKMSEKGTSTHVIT^192^) are recognized by IgG antibodies from tested sera. Within P4 amino acid sequence there are none of the in silico predicted T- or B-cell epitopes, while P8 contains one full (MSEKGTS) and a partial (HVIT) of in silico predicted B-cell epitopes. Interestingly, the highest immunoreactivity was noticed for umbilical cord blood sera in comparison to sera collected from CDI patients. Moreover, P4 and P8 show noticeably higher immunoreactivity with the healthy volunteers’ sera which may indicate that in this group there were people who were never exposed to CDI albeit had antibodies against CD antigens.

Particularly interesting is that the overall profile of peptides immunoreactivity with umbilical cord blood sera is related to the profile obtained for sera from the CDI patients’ group. This is not an obvious observation since it would rather be expected to obtain a profile of immunoreactivity similar to a healthy group.

Additionally, we examined the possible autoimmunoreactive activity of P4 and P8 by a search in IEDB database. This online tool allowed to check whether selected sequences occur as epitopes in other than CD organisms or were shown to have autoimmunoreactive properties. Results show no relation. Furthermore, P4 and P8 were also checked by HMMER web server used to search for the homology. A little homology was found for P8 (^177^LREKMSEKGTSTHVIT^192^) in eukaryote *Nematostella vectensis* (sequence ^4^EKCSKYGISTRLLT^17^, e-value: 0.0058). It is better known as the starlet sea anemone and is one of the laboratory model organism. As this organism is not used in a food nor any other field of industry, there is a small chance for cross-reactivity with its proteins. 

### 3.5. Characterizing the Epitopes of Peptidase M24

**Figure 4:**
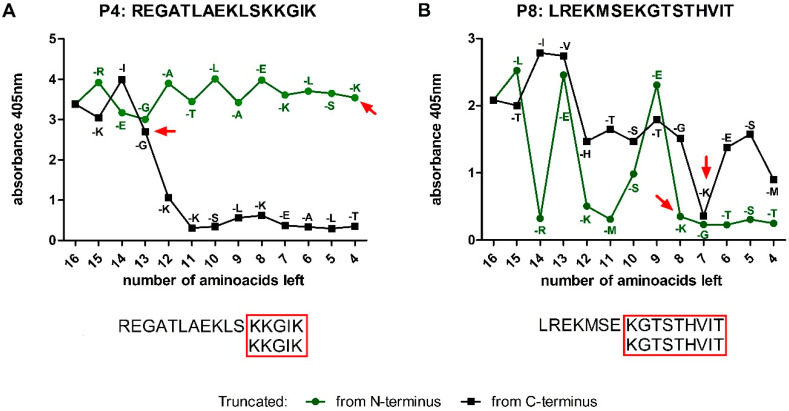
Results of ELISA with truncated from N- and C-terminus peptides mapped with the use of CDI patients’ sera (dilution 1:1000), detected with anti-human IgG secondary antibodies conjugated with AP and colorimetric reaction was developed with AP Yellow Substrate. Red arrows indicate amino acids which absence caused a strong decrease in immunoreactivity. (**A**) The minimal epitope found for P4 (^131^KKGIK^135^); (**B**) the minimal epitope for P8 (^184^KGTSTHVIT^192^).

Subsequently, in order to determine the shortest immunoreactive sequence (epitope), a set of truncated peptides was synthesized on pins. Selected peptides were shortened by one amino acid either from C- or N-end. Next, their immunoreactivity was tested with ELISA using CDI patients’ sera. ^131^KKGIK^135^ (E4) sequence turns out to be the epitope for P4, as well as sequence ^184^KGTSTHVIT^192^ (E8) for P8 (Figure 4A,B).

**Figure 5:**
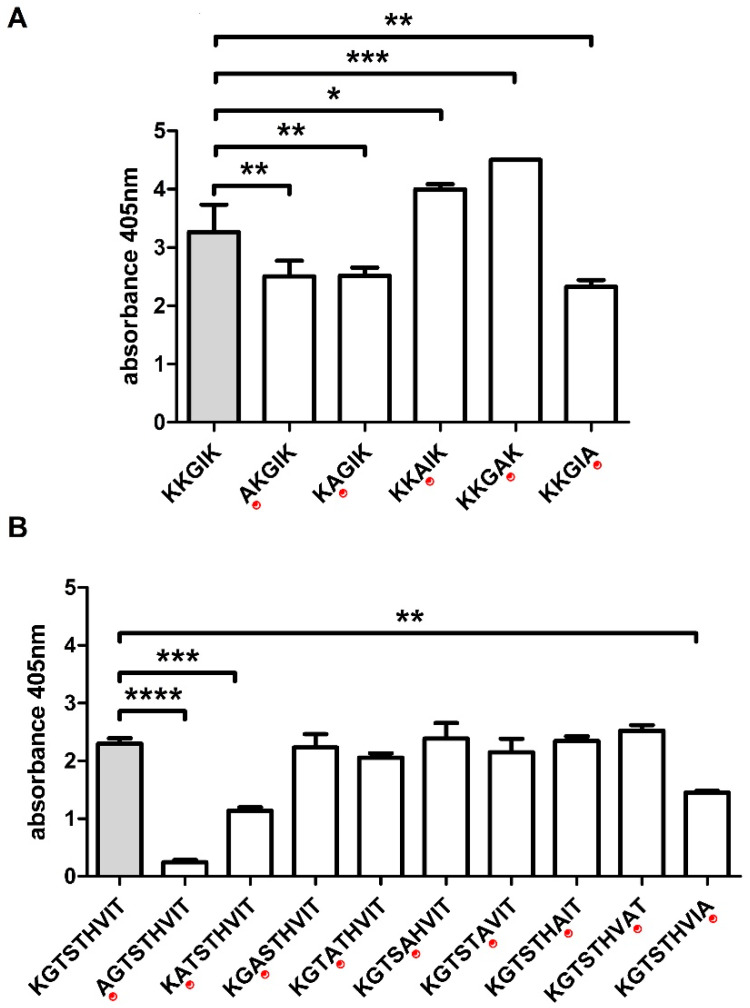
Characteristic of E4 and E8 functional epitopes. (**A**) presents “alanine walk” performed for ^131^KKGIK^135^. (**B**) presents “alanine walk” performed for ^184^KGTSTHVIT^192^. ELISA was repeated at least three times with the use of CDI patients’ sera (dilution 1:1000), detected with anti-human IgG secondary antibodies conjugated with AP, and colorimetric reaction was developed with AP Yellow Substrate. Data are shown with mean and ±SD. 1-way ANOVA and Dunnett’s multiple comparisons test were performed and significant differences were calculated (**** *p* ≤ 0.0001, *** *p* ≤ 0.001, ** *p* ≤ 0.01, * *p* ≤ 0.05).

Moreover, an additional synthesis was performed to determine amino acids that were crucial for antibody binding in each of the above epitopes (functional epitopes). For this purpose, every subsequent amino acid in E4 and E8 was substituted with alanine (so called “alanine walk”). ELISA with sera from CDI patients shows that Lys1, Lys2, and Lys5 from E4 (Figure 5A), as well as Lys1, Gly2, and Thr9 from E8 (Figure 5B), are necessary for their recognition by the antibodies in tested sera since their substitution with alanine causes a drop in the peptide immunoreactivity.

**Figure 6:**
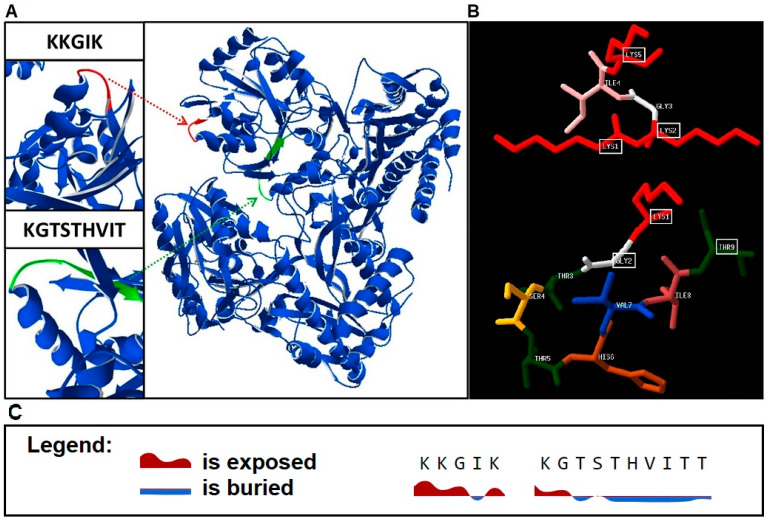
Localization of epitopes of M24 protein. (**A**) Localization of epitopes E4 (^131^KKGIK^135^) and E8 (^184^KGTSTHVIT^192^). (**B**) The structures of E4 (above) and E8 (below) epitopes obtained by the PEP-FOLD3 and visualized by PDB viewer. (**C**) Results for solvent accessibility analysis of E4 and E8 minimal epitopes obtained by NetSurfP-2.0 with a 25% threshold.

The above results enabled the selection of two epitopes E4 (^131^KKGIK^135^) and E8 (^184^KGTSTHVIT^192^) that were proceeded to analysis by PEP-FOLD3 online tool. Protein model analysis shows that E4 is located in a loop and exposed, while E8 might be buried (Figure 6A). Bioinformatics analysis of peptide structure shows that both peptides adopt a secondary structure that corresponds to their predicted conformation in the native protein (Figure 6B). Moreover, the good solvent accessibility of the E4 epitope is confirmed with the use of online tools, while E8 is buried in the protein structure (Figure 6C).

### 3.6. Synthesized Peptide E4 in Free Form Maintains Its Immunoreactivity

**Figure 7:**
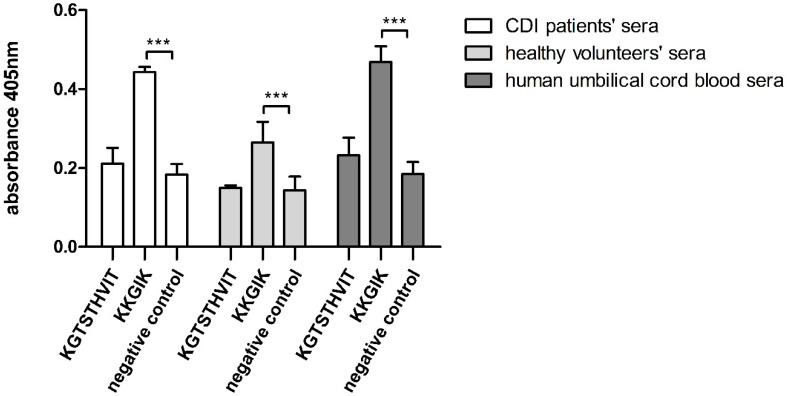
Immunoreactivity analysis of epitopes synthesized in a free form. Streptavidin-coated plates were coated with biotinylated peptides in carbonate buffer (pH = 9.5) in a concentration of 10 μg/mL. ELISA was performed using CDI patients’ sera, healthy volunteers’ sera or human cord blood sera (dilution 1:1000), secondary antibodies anti-human IgG conjugated with AP, and AP Yellow Substrate was used for color reaction. Assay performed in triplicate. Data are shown with mean and ±SD. 2-way ANOVA and Bonferroni posttest were performed. All differences were statistically significant (*** *p* ≤ 0.001).

In the next step we confirmed immunoreactivity of epitopes E4 and E8 that were synthesized in a free form as biotinylated peptides. For this purpose, the ELISA assay was carried out with the use of the three groups of pooled sera described previously (umbilical cord blood, healthy individuals, patients with acute CDI) and streptavidin-coated 96-well plates. Immunoreactivity of the ^131^KKGIK^135^ epitope was 2.5 times higher (Figure 7) compared to the control and ^184^KGTSTHVIT^192^ epitope in ELISA with umbilical cord blood and CDI patients’ sera. At the same time, the level of binding of antibodies from the healthy volunteers’ sera against ^131^KKGIK^135^ was noticeable and statistically significant but weaker than the other tested sera. Based on the obtained results, we decided to focus on the ^131^KKGIK^135^ epitope in further analysis.

### 3.7. KKGIK Conjugated with BSA Induce IL-6 Production by TC-1 Cell Line

**Figure 8:**
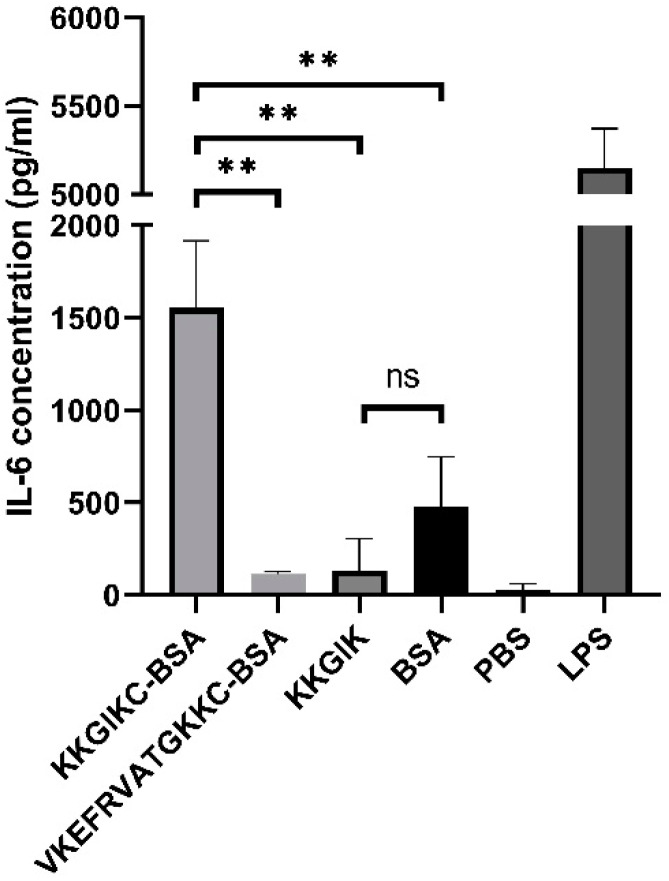
Stimulation of TC-1 epithelial cells with conjugate KKGIKC-BSA (10 μg/mL), BSA (10 μg/mL), KKGIK (10 μg/mL), VKEFRVATGKKC-BSA (10 μg/mL), and LPS (1 μg/mL, positive control). The KKGIKC-BSA induced significantly higher IL-6 production as compared to KKGIK peptide and BSA alone (** *p* ≤ 0.05). IL-6 level was quantified using IL-6 ELISA Kit. Data are shown with mean and ±SD. Unpaired t-tests were performed.

An epithelial cell line TC-1 was used to assess the cellular response to the KKGIKC-BSA, VKEFRVATGKKC-BSA, BSA, and KKGIK peptide alone stimulation. LPS was used as a positive control. After 24 h of incubation, cell culture supernatants were tested by ELISA to determine the level of IL-6 (Figure 8). The results indicate a significant increase in IL-6 levels after stimulation with the KKGIKC-BSA conjugate, compared to the VKEFRVATGKKC-BSA, BSA, and KKGIK control. Noteworthy, this effect is not caused by cytotoxicity of the conjugate since it does not influence cells viability (data not shown). Moreover, the LAL assay revealed that the endotoxin levels were below 0.1 EU per 1 µg of pure conjugate. To sum up, we show that the KKGIKC-BSA conjugate activates the cellular response by stimulating IL-6 production.

## 4. Discussion

Based on recent epidemiological data, the growing threat of CDI is eminent. The search for alternative ways to deal with this pathogen is driven by increasing CD antibiotic resistance, as well as rapidly growing group of patients at risk, since CD has emerged to infect younger people (less than 60 years old) and people without the history of antibiotic treatment [1,58]. Prototype vaccines focused on the neutralization of CD toxins and are giving promising results but may not eradicate colonization of the intestine by CD [11,29]. Our study approach is focused on finding epitopes of CD proteins (other than toxins), which could be a better solution for the CD problem [21,34]. We washed off the surface proteins of the CD using the 1 M LiCl method and evaluated their immunoreactivity by Western blotting with umbilical cord blood. In our research, we focused on bacterial surface proteins, since their external localization makes them easily accessible for immune system during pathogen invasion. However, within this study, we identified a protein that is thought to have intracellular localization (M24), despite of using a protocol designated for surface protein isolation. Nevertheless, there are many proteins that had been previously known to be localized inside the cell, but their new localizations and functions were noticed [59,60,61]. This means that M24 peptidase can be one of the moonlighting proteins and could also be localized on the bacterial cell surface. Overall, localization of peptidase M24 should be further evaluated. 

The study presented here is a part of a project concerning new *Clostridioides difficile* immunoreactive antigens that could be used as antigens’ vaccine in the future (NSC Poland, 2016/21/B/NZ6/02286). Some of our study has already been published [21,34]. In our analysis, we search for new anti-CD vaccine targets using CDI patient sera and umbilical cord blood sera. Since antibody transfer from mother to the fetus is a selective process we, and others, recognize cord blood sera antibodies as conceivably highly protective [21,62,63]. M24 was one of the proteins recognized by umbilical cord blood antibodies and it prompted us to look for its epitopes. Similarly like aminopeptidases from human or *Escherichia coli* (strain K12), CD peptidase M24 uses Mn^2+^ as a cofactor and is likely to play a role in releasing any amino acid located on N-terminus of peptide or polypeptide [39,64]. Since the M24 peptidase model has not been created so far, we decided to make one by using the SWISS-MODEL online software. We obtained a model that is a homodimer, and we used it for further studies. 

A potential application of the peptidase M24 homologue was already described [65]. M24 was one of extracellular proteins from another *Clostridium* species - *Clostridium perfringens* (CP). Sengupta et al. suggested the use of the amino acids metabolizing enzyme (in this case, M24/M37 family peptidase) as a diagnostic marker for the detection of CP in food. We, however, chose to analyze the immunotherapeutic aspect of this protein and more precisely, its epitopes. As far as we know, there are no more scientific reports of this protein.

The mapping of M24 sequence revealed two peptides that showed considerably higher immunoreactivity compared to the other synthesized peptides which were in silico predicted to contain epitopes. These two peptides were P4 (^120^REGATLAEKLSKKGIK^135^) and P8 (^177^LREKMSEKGTSTHVIT^192^). They were strongly recognized by antibodies from the umbilical cord blood sera, indicating an intensified transmission of anti-P4 and anti-P8 antibodies from the mother to the fetus. In addition, pooled sera from CDI patients exhibited high levels of anti-P4 and P8 antibodies. In healthy volunteers’ sera, in turn, unexpected high response may be caused by asymptomatic CD infection or contact with its cross-linking antigens. These data are consistent with what was previously published [3,4,21].

Comprehensive characteristic of immunoreactive peptides P4 (^120^REGATLAEKLS**KKGIK**^135^) and P8 (^177^LREKMSE**KGTSTHVIT**^192^) led to the designation of epitopes localized in these peptides (Figure 4A,B) **^131^KK**GI**K^135^** (E4) and ^184^**KG**TSTHVI**T**^192^ (E8), respectively. Three lysins (K1, K2, and K5) were shown to form the functional epitope in E4. In the structural model obtained by the PEP-FOLD3 software, it is clearly visible how the side chains of K1 and K2 are exposed to the outside of the peptide, creating a docking platform for specific antibodies. In the case of E8, K1, G2, and T9 were found to be essential for antibody binding. K1 is especially important, since its substitution with alanine led to a rapid decrease of E8 immunoreactivity. Lysine is a positively charged amino acid and is extremely important in molecular interactions. In the case of an antibody-antigen binding, it is able to strengthen the connection through hydrogen bonds or electrostatic forces [66,67]. In 2018, Fukunaga et al. showed that the introduction of lysine to the Fab region of an antibody improves its affinity to the given antigen [68]. In turn, Thommesen et al. pointed out the key role of lysine in the human IgG3 for the complement activation [69]. The alignment of E4 and E8 sequences with the peptidase M24 homologues from other organisms showed that these epitopes are distinctive for CD strains and do not appear in proteins from other species (Tables S1–S4). Immunoreactivity analysis completed on E4 and E8 synthesized in a free form showed significantly higher immunoreactivity of E4 as compared to E8 and E4 was further tested. The difference in E8 immunoreactivity, comparing to the pin-bound peptide, may be caused by a dissimilar spatial conformation adopted by unbound peptide compared to the pin-bound one. As a result, this change may affect antibody recognition and binding.

Since the short peptides are rather weak immunogens and sensitive to enzymatic degradation, therefore, they need to be conjugated to a protein carrier [70,71]. Carrier protein facilitates the transport of conjugate to the cell and boosts the cytokine response [70,71,72]. For this reason, E4 (^131^KKGIK^135^) was conjugated to BSA. We tested the immunomodulatory effect of the KKGIKC-BSA by stimulation of an epithelial cell line. The choice of epithelial cell line corresponds with the idea of future preparation of potential intranasal-delivered mucosal vaccine against CD. Since we are dealing with a pathogen that infects mucosal surfaces, it is crucial to induce not only the systemic but also the mucosal immune response that will act at the first step of CD infection. This kind of response will prevent the development of the full spectrum of disease symptoms. Standard vaccines, administered parenterally, rarely induce a proper mucosal immune response [73]. Moreover, the existence of “common mucosal immunity” indicates that stimulation of the nasal mucosa will result in antibody production in the intestinal mucosa [74]. Upon the stimulation with the KKGIKC-BSA conjugate, cells produced considerable amounts of IL-6. The IL-6 is a cytokine that plays a pleiotropic pro- and anti-inflammatory functions [75,76]. It was previously shown that it can be expressed by epithelial cells and distributed locally or secreted to the serum [77]. The presence of the IL-6 alerts the immune system about the threat and indicates the immune response and maturation of immune cells. The obtained effect of IL-6 stimulation comes from the KKGIKC part of the conjugate since BSA or peptide alone induce much lower levels of IL-6. Therefore, we suggest that in the KKGIKC-BSA conjugate, the role of BSA is to make the peptide more “visible” for the cells, probably by facilitation of its engulfment as described by others [71,72]. In the cell line stimulation experiment, we were also treating TC-1 cells with irrelevant VKEFRVATGKKC-BSA conjugate derived from a different CD protein (Figure 8). Since it was not able to induce production of IL-6 in epithelial cells and showed no significant differences compared with the results obtained for the BSA, we conclude that the conjugation process *per se* didn’t influence obtained data. That is why we claim that the conjugate activity is caused by the KKGIKC peptide. It is possible that the presence of the carrier protein increased both peptide accessibility for the cells and its resistance to degradation [70,71]. ^131^KKGIK^135^ is probably recognized by the cell through unidentified receptors, which in result leads to the activation of the immune response. This peptide might have an attractive self-adjuvanting effect and requires additional studies. Self-adjuvants are antigens that do not need an addition of extraneous adjuvants in order to be immunogenic. Such antigens based on peptides are already known [78,79]. The self-adjuvanting properties of ^131^KKGIK^135^ need to be confirmed by in vivo studies.

We chose BSA as a carrier protein since it is widely applied vaccine preparation. Miyairi et al. produced a synthetic vaccine consisting of BSA conjugated with 3-oxododecanoyl-L-homoserine lactone-protein directed against *Pseudomonas aeruginosa*. It induced specific antibodies in mice and block the host inflammatory response by reducing TNF-α levels [80]. Similarly, Laverde et al. were working on the conjugation of BSA with synthetic teichoic acid fragment derived from *Enterococcus faecalis* 12030 (WH7-BSA) that induced specific and opsonic antibodies against this pathogen [81]. There are multiple advantages of using synthetic, peptide-conjugate based vaccines. Mainly, the exact sequence of immunizing antigen is known, thereby reducing risk of auto- and cross-reactivity reactions after immunization. Additionally, during making this type of preparation, there is no need to work with the whole pathogen which reduces the possibility of contaminating the vaccine with the living microorganism [70,71,72]. A synthetic vaccine that has reached the stage of clinical trials is directed against the protozoan causing malaria. The safety and immunogenicity of this vaccine has been confirmed [82]. The effects of studies of some other authors also reached clinical trials [83].

Immunocompetent cells do not recognize the whole protein but only its intrinsic antigenic determinants. ^131^KKGIK^135^ sequence is relatively short as an epitope. However, literature data show mostly short epitopes (up to 4 amino acids), which are often found in the surrounding area of free loops or beta-sheet structures [84]. To prevent short epitopes from degradation, their length can be increased by adding amino acids on both sides (flanking) of the peptide, cyclizing the peptide or adding unnatural amino acids to its sequence [71,85]. 

The epitope ^131^KKGIK^135^ determined in our research, in combination with the carrier protein, fulfills the criteria for a good vaccine antigen: (1) it is immunoreactive, (2) it has a modulatory effect on the immune system by stimulating the secretion of IL-6, (3) its sequence is conserved within CD strains, and (4) it is safe, what was confirmed by the analysis of cross-reactivity. These properties are planned to be verified by us in in vivo studies. Since currently there is no vaccine built on other chemically synthesized peptides available on the market, the extensive research on this topic is warranted. Razim et al. identified three epitopes in a CD the Cwp22 surface protein—^54^EFRVAT^59^, ^201^KVNGKM^206^, and ^268^WQEKNGKKYY^277^ [34]. Jarząb et al. identified the RYDERY epitope that might be a good candidate for an anti-enterobacterial vaccine against *Shigella flexneri* [52]. 

Preexisting strong immune response at the first contact with CD relates to lower likelihood of disease symptoms. In our research, in silico and in vitro studies of peptidase M24 led to the selection of the ^131^KKGIK^135^ as a novel CD epitope. It has a potential to be used in developing synthetic vaccines or to create the therapeutic antibodies that can succor the antibiotic treatment of infected patient. Moreover, we suggest that a combination of our epitope and toxin-based antigens would be equally good solution for the CD challenge. This preparation may efficiently prevent the CD colonization of human intestine [11,29]. To sum up, we propose the use of ^131^KKGIK^135^ as a suitable antigen to create the robust vaccine.

## Supplementary Materials

The following supplements are available online at https://www.mdpi.com/2073-4409/9/5/1146/s1, **Table S1**: Peptidase M24 homologues list among genus *Clostridium*, their length and the identity level with the reference protein CBE05263.1; **Table S2**: Human peptidase M24 homologues, its length and the identity level with the reference protein CBE05263.1; **Table S3**: Peptidase M24 homologues list among human flora, its length and the identity level with the reference protein CBE05263.1; **Table S4**: Peptidase M24 homologues list among *Clostridioides difficile* strains, its length and the identity level with the reference protein CBE05263.1; **Figure S1**: Conservativeness analysis of the KKGIK epitope within the human physiological flora carried out using the Clustal Omega online tool; **Figure S2**: Conservativeness analysis of the KKGIK epitope within human proteins carried out using the Clustal Omega online tool; **Figure S3**: Conservativeness analysis of the KGTSTHVIT epitope within the human physiological flora carried out using the Clustal Omega online tool; **Figure S4**: Conservativeness analysis of the KGTSTHVIT epitope within human proteins carried out using the Clustal Omega online tool.
