# Supplementary material for: The Bioinformatic and In Vitro Studies of Clostridioides Difficile Aminopeptidase M24 Revealed the Immunoreactive KKGIK Peptide
Source: Cells. 2020 May 7;9(5):1146. doi: 10.3390/cells9051146 (PMC7291276; doi:10.3390/cells9051146)
Supplement: Supplementary file 1 [file cells-09-01146-s001.pdf]

Table S1. Human M24 peptidase homologues, its length and the identity level with the reference CD R20291 protein M24 peptidase (accession NCBI no. CBE05263.1)

Protein name	Reference number	Length (aa)	Identity (%)
1. Xaa-Pro aminopeptydase 1	UniProtKB: Q9NQW7	597	40
2. Xaa-Pro aminopeptydase 2	UniProtKB: 043895	674	35

Table S2. Peptidase M24 homologues list among human microflora, its length and the identity level with the reference CD R20291 protein M24 peptidase (accession NCBI no. CBE05263.1)

Genus	Reference number	Length (aa)	Identity (%)
1. *Enterococcus faecalis* (2)	GenBank: KGQ72307.1	925	86
2. *Aerococcus viridans*	GenBank: AMC00482.1	459	63
3. *Eubacterium sp. 3_1_31*	NCBI Reference Sequence: WP_008689739.1	596	56
4. *Ruminococcus sp. DSM 100440* (2)	GenBank: KYG86539.1	596	56
5. *Ruminococcus sp. DSM 100440* (1)	GenBank: KYG86232.1	595	54
6. *Enterococcus faecalis* (1)	GenBank: OCK15859.1	466	50
7. *Bacteroides fragilis*	NCBI Reference Sequence: WP_053874255.1	592	43
8. *Bacteroides sp. 3_1_40A*	GenBank: EFV68935.1	593	43
9. *Bacillus subtilis*	NCBI Reference Sequence: WP_025709700.1	577	37
10. *Staphylococcus epidermidis*	GenBank: OBZ51921.1	353	33
11. *Staphylococcus aureus*	GenBank: KII20940.1	353	32
12. *Streptococcus mutans*	NCBI Reference Sequence: WP_024785215.1	354	29
13. *Streptococcus anginosus*	NCBI Reference Sequence: WP_025271618.1	353	28
14. *Escherichia coli ATCC 8739*	GenBank: ACA79760.1	443	26
15. *Streptococcus salivarius*	NCBI Reference Sequence: WP_002889263.1	353	25
16. *Streptococcus pyogenes*	NCBI Reference Sequence: WP_042361523.1	357	25
17. *Lactobacillus sp. ASF360*	NCBI Reference Sequence: WP_004045994.1	369	24

18. *Bifidobacterium animalis subsp. lactis*	GenBank: AJD88894.1	523	23

Table S3. Peptidase M24 homologues list among genus *Clostridioides* their length and the identity level with the reference CD R20291 protein M24 peptidase (accession NCBI no. CBE05263.1).

Genus	Reference number	Length (aa)	Identity (%)
1. *Clostridium dakarense*	NCBI Reference Sequence: WP_042277847.1	596	73
2. *Clostridium bornimense*	NCBI Reference Sequence: WP_044036809.1	596	63
3. *Clostridium botulinum* (2)	GenBank: KON10815.1	597	54
4. *Clostridium intestinale*	NCBI Reference Sequence: WP_021803961.1	592	52
5. *Clostridium perfringens*	GenBank: ALG48248.1	591	51
6. *Clostridium sp. DL-VIII*	GenBank: EHI97030.1	591	50
7. *Clostridium neonatale*	NCBI Reference Sequence: WP_058293168.1	591	50
8. *Clostridium homopropionicum*	NCBI Reference Sequence: WP_052220382.1	592	50
9. *Clostridium saccharoper-butylacetonicum*	GenBank: AGF53900.1	591	49
10. *Clostridium baratii*	NCBI Reference Sequence: WP_054200563.1	591	49
11. *Clostridium colicanis*	NCBI Reference Sequence: WP_061858919.1	590	48
12. *Clostridium sartagoforme*	NCBI Reference Sequence: WP_051993126.1	578	48
13. *Clostridium celatum*	NCBI Reference Sequence: WP_040418690.1	587	48
14. *Clostridium butyricum*	GenBank: KHD15142.1	415	33
15. *Clostridium tetani E88*	GenBank: AAO36137.1	359	28
16. *Clostridium sordellii*	GenBank: CEQ29662.1	408	27

Table S4. Peptidase M24 homologues list among *Clostridium difficile* strains, its length and the identity level with the reference CD R20291 protein M24 peptidase (accession NCBI no. CBE05263.1).

Strain	Reference number	Length (aa)	Identity (%)
1. *Peptoclostridium difficile CD196*	GenBank: CBA64059.1	597	100

2. *Clostridioides difficile 630* (1)	GenBank: CAJ69146.1	597	99
3. *Clostridioides difficile ATCC 9689 = DSM 1296*	GenBank: AKP43213.1	597	99
4. *Clostridioides difficile DA00210*	GenBank: EQH18240.1	597	99
5. *Clostridioides difficile E12*	GenBank: CCL82046.1	597	99
6. *Clostridioides difficile T15*	GenBank: CCL22579.1	597	99
7. *Clostridioides difficile CD69*	GenBank: EQE84866.1	597	98
8. *Clostridioides difficile E10* (2)	GenBank: CCK98675.1	597	98
9. *Clostridioides difficile 630* (2)	NCBI Reference Sequence: YP_001088999.1	354	27
10. *Clostridioides difficile T10*	GenBank: CCL88333.1	354	27
11. *Clostridioides difficile E1*	GenBank: CCK94965.1	354	25
12. *Clostridioides difficile E10* (1)	GenBank: CCK98905.1	354	25
13. *Clostridioides difficile T22*	GenBank: CCL14867.1	354	25

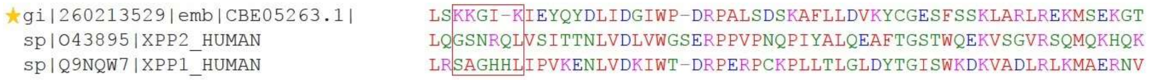

Figure S1. Conservativeness analysis of the KKGIK epitope within the human physiological microflora carried out using the Clustal Omega online tool. The star indicates the *Clostridium difficile* R20291 peptidase M24 sequence.

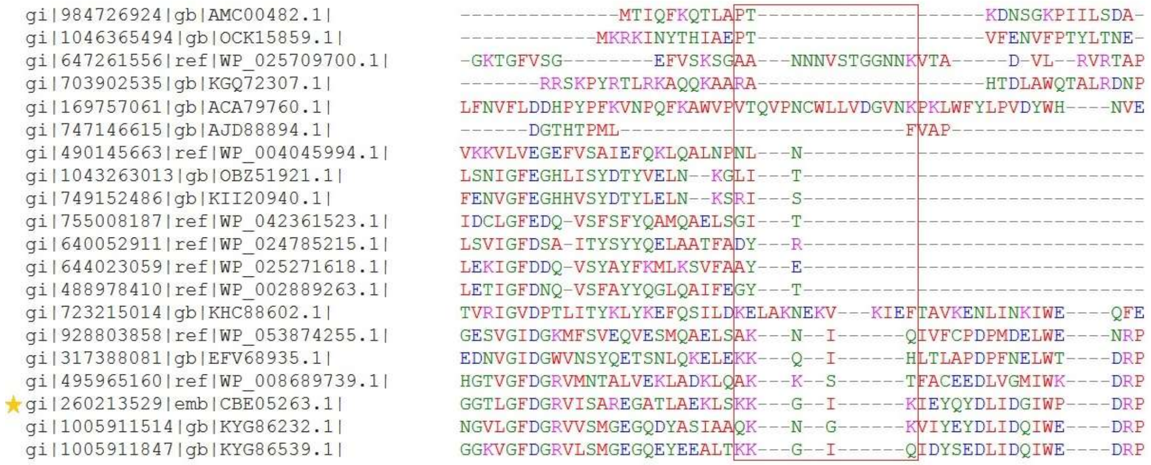

Figure S2. Conservativeness analysis of the KKGIK epitope within human proteins carried out using the Clustal Omega online tool. The star indicates the *Clostridium difficile* R20291 peptidase M24 sequence.

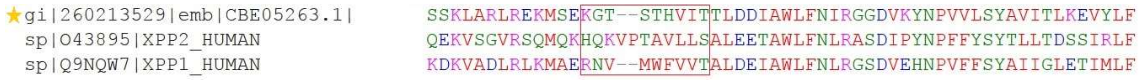

Figure S3. Conservativeness analysis of the KGTSTHVIT epitope within the human physiological flora carried out using the Clustal Omega online tool. The star indicates the *Clostridium difficile* R20291 peptidase M24 sequence.

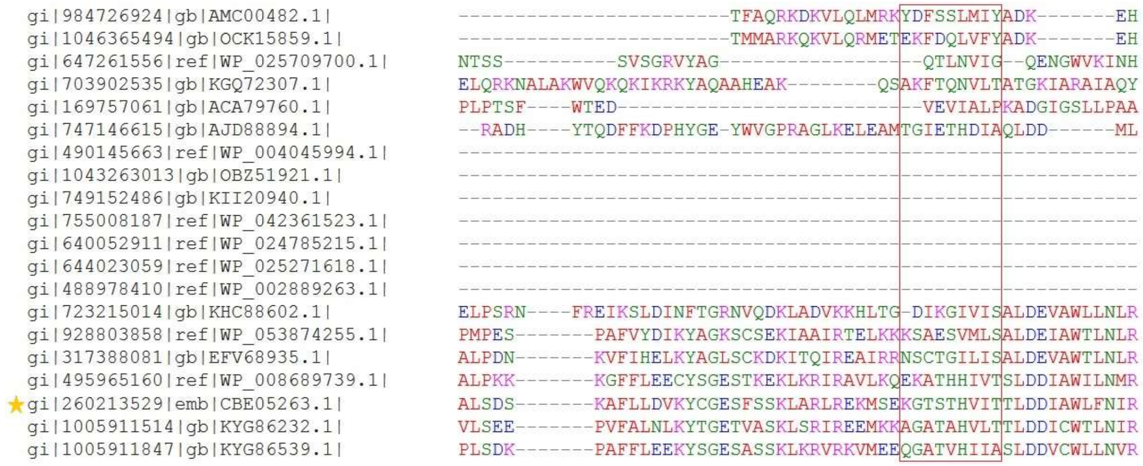

Figure S4. Conservativeness analysis of the KGTSTHVIT epitope within human proteins carried out using the Clustal Omega online tool. The star indicates the *Clostridium difficile* R20291 peptidase M24 sequence.
